# Regulatory role of N6-Methyladenosine on skeletal muscle development in Hu sheep

**DOI:** 10.3389/fgene.2024.1449144

**Published:** 2024-08-21

**Authors:** Junfang Jiang, Liangyong Guo, Xin Huang, Kaizhi Zheng, Sangang He, Huili Shan

**Affiliations:** ^1^ Institute of Animal Husbandry and Veterinary, Zhejiang Academy of Agricultural Sciences, Hangzhou, China; ^2^ Huzhou Agricultural Science and Technology Development Center, Institute of animal Science, Huzhou, China; ^3^ Huzhou Key Laboratory of Innovation and Application of Agricultural Germplasm Resources, Huzhou, China

**Keywords:** Hu sheep, longissimus dorsi muscle, skeletal muscle development, m6A modification, MeRIP-seq

## Abstract

N6-Methyladenosine (m6A) RNA modification plays an essential role in many biological processes. To investigate the regulatory role of m6A on the skeletal muscle development in Hu sheep, this study took newborn Hu sheep (b_B Group) and six-month-old Hu sheep (s_B Group) as the objects. MeRIP-seq and RNA-Seq analysis techniques were used to detect differentially methylated genes (DMGs) and differentially expressed genes (DEGs) in the longissimus dorsi muscle of Hu sheep at different months of age. Then, conjoint analysis was further employed to screen for key genes involved in skeletal muscle development that are modified by m6A and expressed by mRNA. According to the results of the MeRIP-seq analysis, there were 285 m6A differentially methylated peaks (DMPs) in total between b_B Group and s_B Group, with 192 significant upregulated peaks and 93 significant downregulated peaks. GO and KEGG analysis revealed that DMGs are mainly enriched in actin-binding, cellular transport, and metabolic pathways. According to the results of the RNA-seq analysis, there were 4,349 DEGs in total between b_B Group and s_B Group, with 2010 upregulated genes and 2,339 downregulated genes. DEGs are found to be mainly enriched in the regulation of actin cytoskeleton tissue, AMPK and FoxO signaling pathways, etc. The conjoint analysis demonstrated that 283 genes were both modified by m6A and expressed by mRNA. Among them, three genes relevant to muscle growth (RGMB, MAPK8IP3, and RSPO3) were selected as candidates for quantitative validation, and the results were in line with the sequencing results. The results mentioned above all suggest that m6A plays a certain role in the skeletal muscle development in Hu sheep.

## 1 Introduction

N6-Methyladenosine (m6A) refers to the methylation modification that occurs on the sixth nitrogen atom of adenine (Wang et al., 2022). m6A methylation is the most common chemical modification of mRNA in eukaryotes, mediated by methyltransferase complexes centered around methyltransferaselike3 (METTL3) and methyltransferaselike14 (METTL14) (Meyer et al., 2012; Liu et al., 2014; Sorci et al., 2018). RNA methylation modification is a regulatory mechanism that controls gene expression in eukaryotic cells (Huang et al., 2020). As a reversible epigenetic modification, m6A is found not only in messenger RNAs but also in non-coding RNAs, which has an impact on the formation of modified RNA molecules and plays an important role in almost all popular biological processes (Huang et al., 2020).

It was found that m6A modification has potential regulatory effects on the growth and development of animal muscle. Deng et al. (2021) performed MeRIP-seq analysis on the skeletal muscle of Haimen goats and resolved the characteristics of m6A modification during muscle development. Zhang et al. (2020) found that m6A methylation and IGF2BP1 play important roles in the regulation of prenatal myogenesis in pig embryos. Ma et al. (2022) found that the m6A gene is mainly involved in regulating longissimus dorsi muscle (LD) differentiation and development in yaks. Chen et al. (2022) found that m6A modification has an impact on ducks’ muscle differentiation by regulating gene expression. Xu et al. (2021) uncovered a transcriptome-wide m6A modification pattern that affects embryonic breast muscle development in Dingan goose. As a valuable native sheep breed in China, Hu sheep are characterized by rapid growth, high reproductive rate, tender, and juicy meat (Zhao et al., 2024). There has been no previous research on the regulatory role of m6A in skeletal muscle development in Hu sheep, this study should be conducted, in order to study the role of m6A modification in Hu sheep and provide a theoretical regulatory mechanism for the growth and development of Hu sheep.

## 2 Materials and methods

### 2.1 Sample collection

Six healthy Hu sheep were selected for this study, three in the newborn (0 day of age) group (b_B Group) and three in the six-month-old (180 days of age) group (s_B Group), all of which were female individuals. The Hu sheep used in the experiment were supplied by Huzhou Yihui Eco-Agriculture Co., Ltd. (Zhejiang, China), whose feeding criteria were environmentally friendly. The test sheep’s longissimus dorsi muscles were extracted after slaughter and promptly kept in liquid nitrogen before being stored at −80°C for future studies. All experimental techniques followed the rules established by the Experimental Animal Management Committee of the Zhejiang Academy of Agricultural Sciences.

### 2.2 Test procedures

#### 2.2.1 RNA extraction and fragmentation

Total RNA was extracted and purified with TRIzol reagent (Invitrogen, United States). 10 ug of total RNA was mixed with RNA Fragmentation Reagents (Invitrogen, United States) for 10 min of reaction at 70°C in a Thermomixer to break the RNA into fragments with a size of approximately 100 nt. The fragmented RNA was precipitated using the ethanol technique.

#### 2.2.2 m6A enrichment

The magnetic beads containing protein A and protein G (Invitrogen, United States) were first cleaned with IP buffer (150 mM NaCl, 10 mM Tris-HCl, pH = 7.5) and then treated for 2 h at 4°C with 5 ug of m6A antibody (Millipore). Second, the magnetic beads were washed twice and resuspended using IP buffer for flipping at 4°C for 4 h, with fragmented RNA added. Next, incubate the magnetic beads for 1 hour at 4°C with m6A competitive eluent after washing the magnetic beads three times with IP buffer. The supernatant containing the eluted m6A RNA was transferred to a new test tube for purification using a reagent of phenol, chloroform, and isoamyl alcohol with a ratio of 125:24:1.

### 2.3 Library preparation

Reverse transcription and library preparation on IP and Input samples were carried out using SMARTer^®^ Stranded Total RNA-seq Kit v2-Pico Input Mammalian User Manual (Takara, JPN). To get the final library, AMPure XP bead [SpeedBead lagnetic Carboxylate lodified Particles (GE, United States)] was employed to choose fragment sizes. After successfully passing the library detection, library pooling was conducted in accordance with the intended sequencing data volume and effective concentration requirements. The Illumina Nova platform was utilized to sequence the libraries, with a strategy of PE150.

### 2.4 Bioinformatics analysis

High-throughput sequencers converted picture data from sequencing fragments into reads using CASAVA base recognition (Ma et al., 2019). Trimmatic (http://www.usadellab.org/cms/?page=trimmomatic) was used to perform quality control on the raw sequencing data, including IP and Input samples, by deleting joints, duplicate sequences, and low-quality sequences in order to obtain CleanData. The obtained CleanData was compared to the genome using hisat2 (Kim et al., 2015) (https://ccb.jhu.edu/software/hisat2/manual.shtml). The bam files obtained from IP and Input samples were subjected to peak calling analysis and differential peak analysis using exomePeak of R Package (Meng et al., 2013; Meng et al., 2014) (https://bioconductor.org/packages/exomePeak). The peak was then annotated using ChIPseeker (Yu et al., 2015) (https://bioconductor.org/packages/ChIPseeker). Finally, motif analysis was performed using HOMER (Heinz et al., 2010) (http://homer.ucsd.edu/homer/motif). The gene assembly and quantification software was StringTie (Pertea et al., 2016) (https://ccb.jhu.edu/software/stringtie) and the quantification method was TPM (Transcripts Per Million mapped reads). DEGs were analyzed using the edgeR of R Package (Meng et al., 2013; Meng et al., 2014) (https://bioconductor.org/packages/exomePeak).

### 2.5 Quantitative real-time PCR

Three genes (RGMB, MAPK8IP3, RSPO3) were screened via the conjoint analysis based on differential m6A-associated genes and DEGs for qRT-PCR analysis. Reverse transcription was conducted on the total RNA using Polestar first cDNA Synthesis Kit (Tiosbio, China). Real-time quantitative PCR was performed using SYBR^®^ Green Realtime PCR Master Mix-Plus (TOYOBO, Japan). The primers were designed using DNAMAN8 with the GAPDH gene of sheep as the internal reference gene (Vorachek et al., 2013). The primer sequences are shown in Supplementary Table S3. Three biological replicates were performed for each sample, and the relative expression was calculated by the 2^−△Ct^ method (Alcaraz-López et al., 2020).

## 3 Results

### 3.1 Data statistics and audit

This study preprocessed the raw data, removing redundant information such as joint sequences and low-quality bases by using Trimmomatic. As shown in Supplementary Table S1, the filtered reads of the six IP libraries were 102,111,742–145,130,626. The filtered reads of the Input libraries were 117,937,062–149,706,464.

### 3.2 Data analysis

The filtered data were compared to the reference genome using hisat2 (Kim et al., 2015). It was found that the proportion of read pairs (Uniq_Rate) of all samples that were accurately matched to a position in the reference genome was more than 54.40%, and the results are shown in Supplementary Table S2.

**FIGURE 1:**
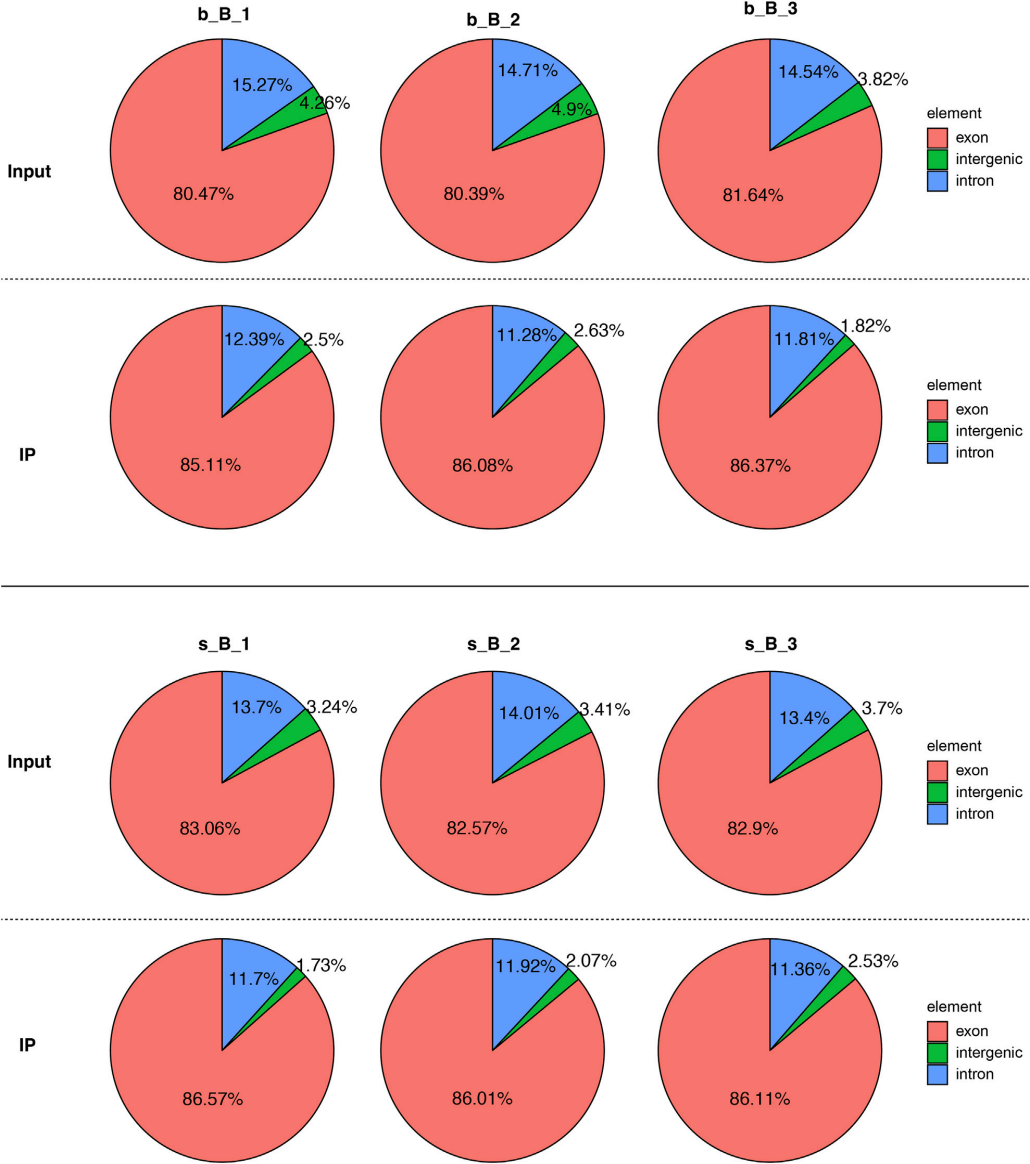
Comparison region distribution of reference genome.

The distribution of reference genome comparison regions showed that the percentage contents of sequenced sequences localized to exon regions were the highest as shown in Figure 1.

### 3.3 Identification of m6A modification sites and analysis of DMGs

**FIGURE 2:**
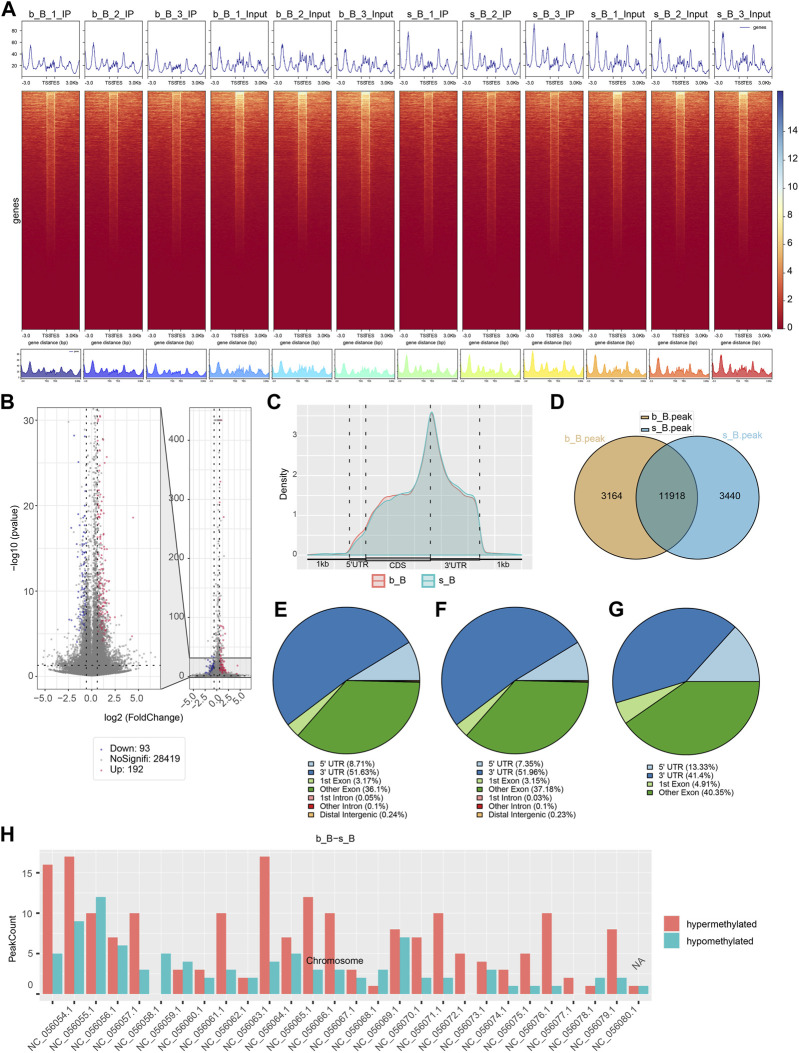
Analysis of m6A modification in the longissimus dorsi muscle of Hu sheep **(A)** Heat map of the enrichment degree of reads near TSS; **(B)** Visualized volcano plot of differentially m6A-modified regions; **(C)** Distribution density of differential m6A modifications across gene elements; **(D)** Veen plots of peak-annotated genomes; **(E,F)** m6A peak distribution of b_B Group and s_B Group; **(G)** Differentially m6A peak distribution of b_B Group and s_B Group; **(H)** Distribution of differential peaks on chromosomes.

The length and location information of the peaks on the genome were determined using the MeRIP-seq data. Reads were found to be abundant near the transcriptome start site (TSS) of the gene. The distribution of reads in the combinable region near the TSS was shown in the form of heatmap plot (as shown in Figure 2A). There were 285 m6A DMPs in total between b_B Group and s_B Group, with 192 being significant upregulated and 93 being significant downregulated (as shown in Figure 2B). The peaks in b_B Group and s_B Group were primarily enriched in the 3′UTR (as shown in Figure 2C). exomePeak analysis found 3,164 and 3,440 specific peaks in b_B Group and s_B Group, respectively, with 11,918 similar peaks between the two groups (as shown in Figure 2D). The peak distributions of the two groups were similar (as shown in Figures 2E, F). The analysis findings revealed that differential peaks were primarily distributed in the 3′UTR (as shown in Figure 2G). Figure 2H demonstrates that differential peaks were found on each chromosome. Table 1 shows the top 20 m6A peaks, with 15 upregulated and 5 downregulated.

**TABLE 1:** Top 20 differential m6A peaks.

Gene name	Peak start	Peak end	diff.log2.fc	Regulation	Peak region	Distance to TSS
COL6A3	3,817,926	3,820,342	0.621	Up	Exon	40,004
ZSCAN20	8,383,009	8,387,910	0.877	Up	Exon	10,907
IPO13	18,589,010	18,591,433	0.78	Up	5′UTR	598
PODN	27,613,790	27,614,299	1.18	Up	3′UTR	22,880
CCN1	62,638,019	62,638,612	0.981	Up	3′UTR	1951
CELSR2	86,532,293	86,538,732	1.14	Up	Exon	2,274
ZNF687	101,198,993	101,200,127	0.996	Up	Exon	3,943
FLAD1	105,206,647	105,207,007	1.16	Up	Exon	2,449
ST3GAL6	164,029,468	164,029,828	−0.975	Down	3′UTR	82,401
PLCL2	275,036,886	275,037,304	0.922	Up	Exon	121,642
ATG4B	272,064	272,333	1.96	Up	3′UTR	15,394
SNED1	660,713	661,552	0.99	Up	3′UTR	79,740
BCAS2	91,947,962	91,949,973	−1.07	Down	3′UTR	11,295
KHDC4	105,943,804	105,944,400	1.23	Up	3′UTR	16,350
ILDR2	118,935,862	118,938,832	1.78	Up	3′UTR	68,571
DCBLD2	164,029,493	164,029,823	−0.962	Down	3′UTR	82,406
ZBTB20	179,896,433	179,896,974	−0.848	Down	3′UTR	64,467
PCYT1A	191,664,961	191,667,956	0.61	Up	3′UTR	41,021
GFM1	229,583,042	229,583,500	−0.774	Down	3′UTR	50,670
DIPK2A	245,504,001	245,522,636	1.2	Up	5′UTR	34

### 3.4 Motif analysis

**FIGURE 3:**
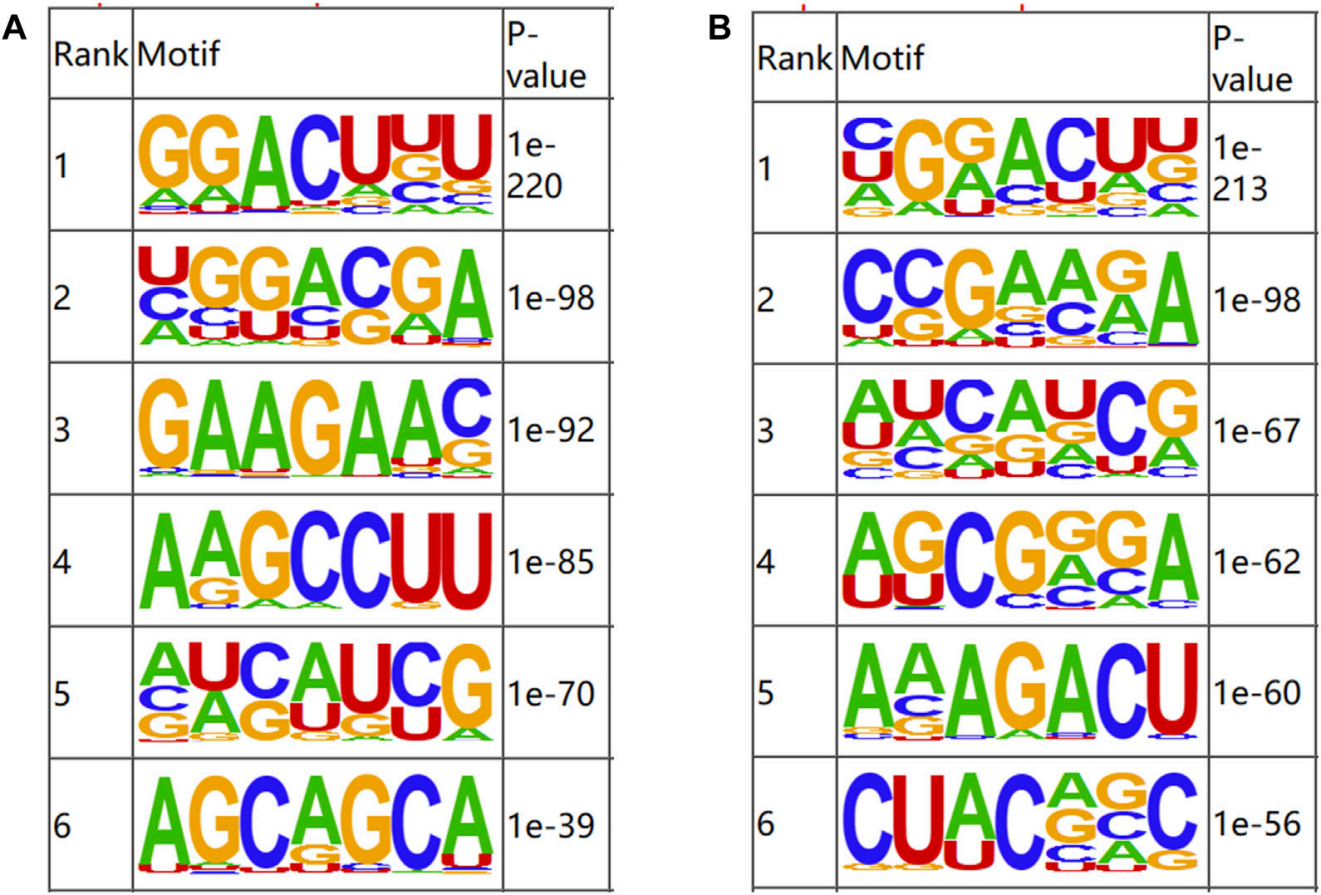
Motifs of m6A-modified regions in b_B Group and s_B Group **(A)** The first six motifs of b_B Group; **(B)** The first six motifs of s_B Group.

OMER (Hansen et al., 2016) was utilized to identify the motifs in the m6A-modified regions. The motifs were ranked according to the value of *P*, with the smaller *P* ranking higher. The results of motif analysis are shown in Figure 3. RRACH, a common motif structure in RNA modification, was present in samples from both groups (where R = A or G; H = A, C or U).

### 3.5 Enrichment analysis of DMGs

**FIGURE 4:**
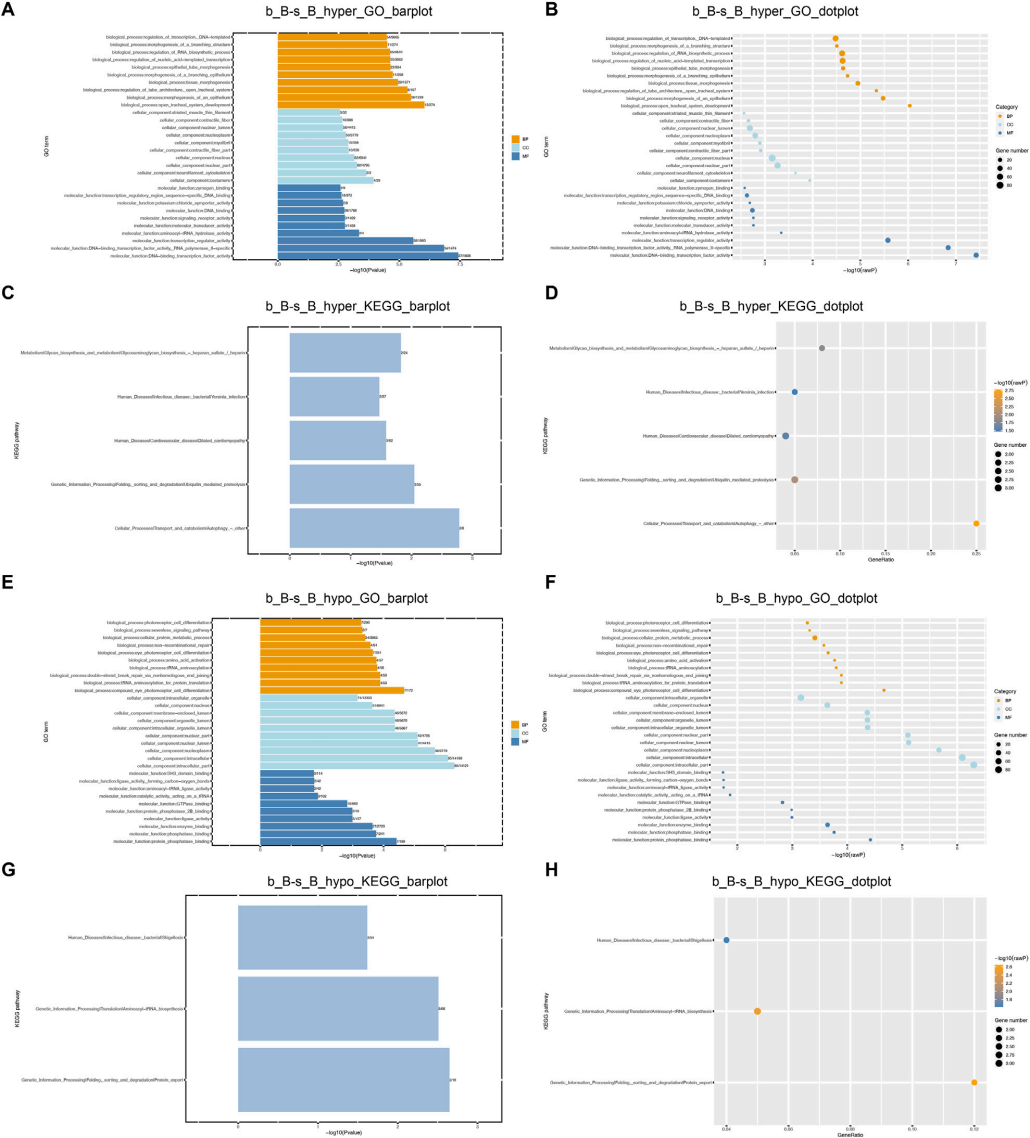
GO and KEGG Enrichment Analysis of DMGs **(A)** b_B-s_B_hyper_GO_barplot; **(B)** b_B-s_B_hyper_GO_dotplot; **(C)** b_B-s_B_hyper_KEGG_barplot; **(D)** b_B-s_B_hyper_KEGG_dotplot; **(E)** b_B-s_B_hypo_GO_barplot; **(F)** b_B-s_B_hypo_GO_barplot; **(G)** b_B-s_B_hypo_KEGG_barplot; **(H)** b_B-s_B_hypo_KEGG_dotplot.

GO and KEGG enrichment analyses were performed on DMGs to analyze their potential functions in the skeletal muscle development of Hu sheep. The enriched GO terms for the DMGs mainly include protein modification process, regulation of macromolecule metabolic process, actin-binding, actin cytoskeleton organization, and structural constituent of muscle. KEGG pathway enrichment analysis showed that DMGs were enriched to the cellular transport, metabolism, autophagy, processing of genetic information, ubiquitin-mediated protective lysis, glycan biosynthesis and metabolism, as shown in Figure 4.

### 3.6 Analysis of DEGs

**FIGURE 5:**
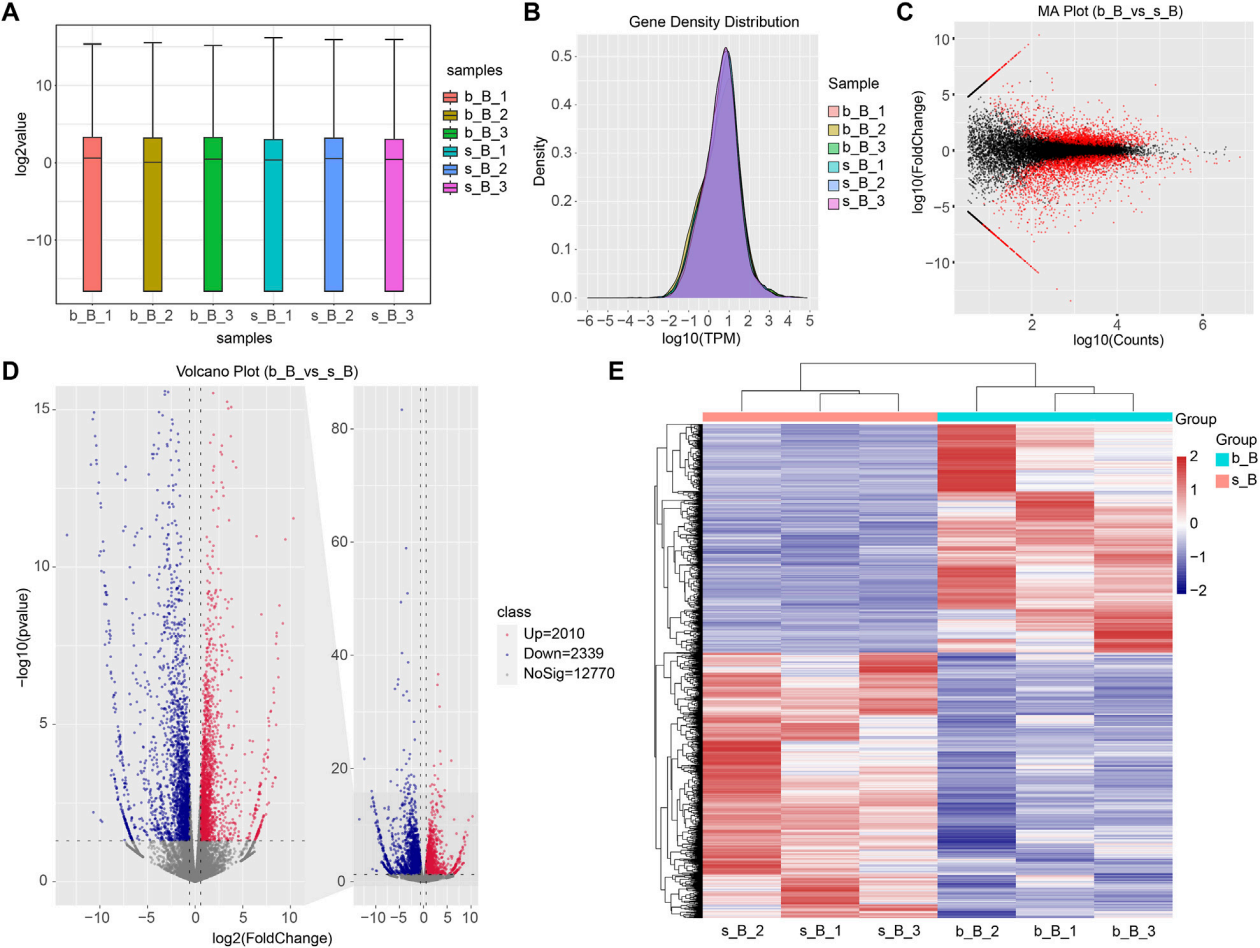
Identification of DEGs **(A)** Gene expression box plot; **(B)** Gene expression density plot for DEGs; **(C)** MA plot for DEGs; **(D)** Volcano plot for DEGs; **(E)** Heat map for DEGs.

The gene expression amount and density were demonstrated in Figures 5A, B. There were 4,349 DEGs detected in total between b_B Group and s_B Group, with 2010 upregulated genes and 2,339 downregulated genes (as shown in Figure 5D). The distribution of DEGs was demonstrated by MA plots, volcano plots, and heat maps, respectively (as shown in Figures 5C–E). Table 2 shows the top 20 DEGs, with 10 upregulated and 10 downregulated.

**TABLE 2:** Top 20 DEGs.

GeneID	Base mean	log2 foldchange	Regulation	*p* value	*p*-adj
A1BG	41.9550	−1.9005	Down	0.0395	0.1521
A4GNT	11.3670	−7.2985	Down	0.0125	0.0682
AAMDC	4,360.6926	−0.7293	Down	0.0405	0.1546
AARS1	6,342.7562	0.7796	Up	0.0010	0.0097
AASDHPPT	2,999.8927	0.8345	Up	0.0061	0.0400
AASS	3,240.6637	−0.6108	Down	0.0220	0.1020
AATF	1,472.4988	1.2338	Up	0.0214	0.1002
ABAT	246.6002	−1.8816	Down	0.0001	0.0015
ABCA10	645.6183	0.6249	Up	0.0289	0.1228
ABCA13	14.3525	−7.6343	Down	0.0002	0.0023
ABCA7	38.3985	−9.0572	Down	0.0000	0.0000
ABCB11	113.0046	−10.6131	Down	0.0000	0.0000
ABCC1	3,928.5716	−0.7248	Down	0.0168	0.0849
ABCC3	251.2373	−1.4250	Down	0.0131	0.0707
ABCC8	392.3889	1.1633	Up	0.0012	0.0111
ABCE1	4,056.6409	0.6891	Up	0.0403	0.1540
ABCF1	8,423.0866	0.6292	Up	0.0051	0.0349
ABCG8	41.7259	8.4901	Up	0.0000	0.0000
ABHD11	265.6319	1.3122	Up	0.0011	0.0106
ABHD14A	317.6643	1.1347	Up	0.0002	0.0026

**FIGURE 6:**
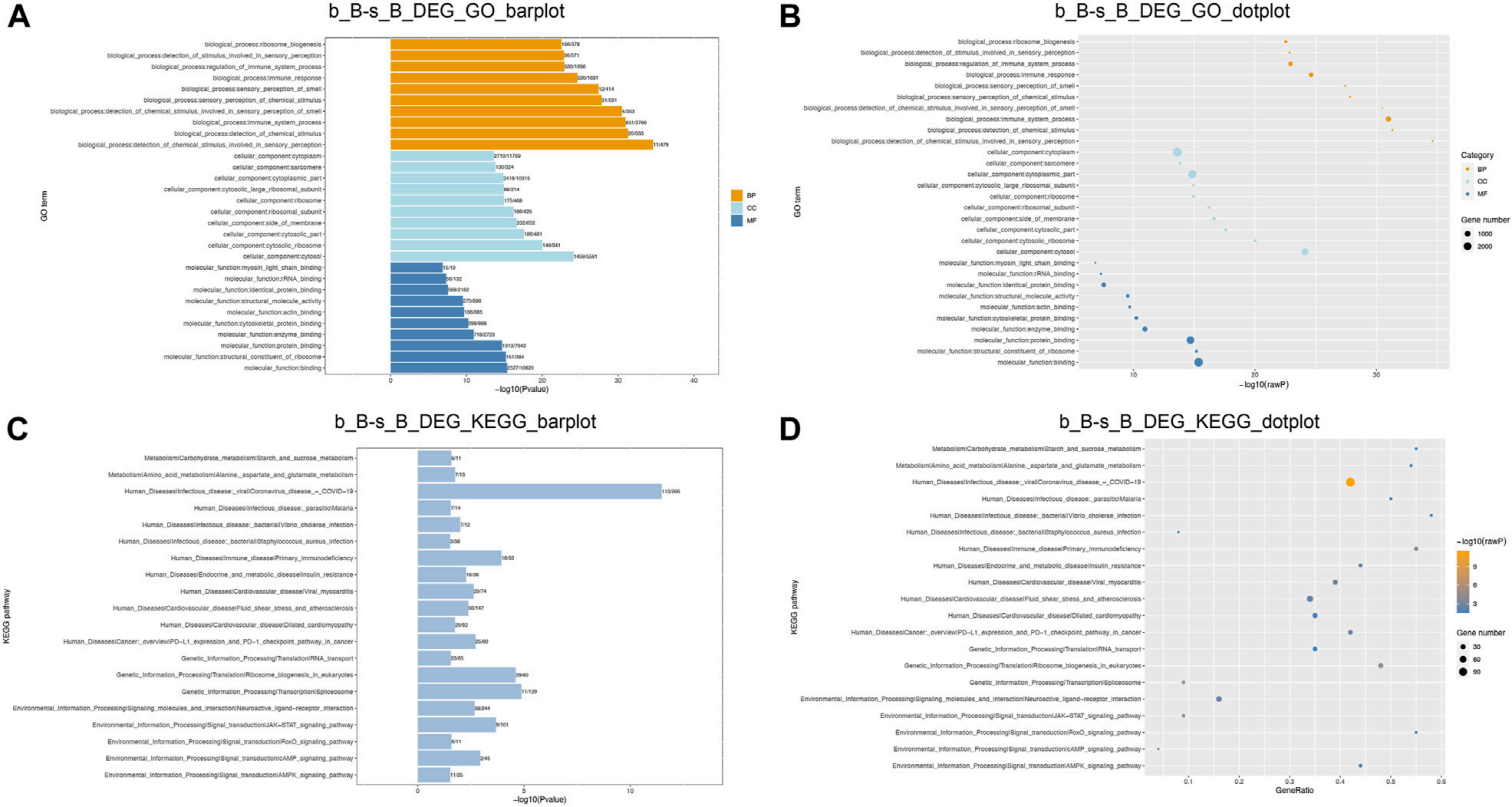
GO and KEGG enrichment analysis of DEGs function **(A,B)** DEGs GO enrichment analysis; **(C,D)** DEGs KEGG enrichment analysis.

GO and KEGG enrichment analyses were conducted to further investigate the functions of DEGs. The enriched GO terms for the DEGs mainly include regulation of muscle cell differentiation, actin cytoskeleton, skeletal muscle tissue growth, and carbohydrate metabolism processes (as shown in Figures 6A, B). KEGG pathway enrichment analysis demonstrated that DEGs showed a significant enrichment to the signaling pathways related to muscle development, including the cAMP signaling pathway, AMPK signaling pathway, FoxO signaling pathway, and JAK-STAT signaling pathway, as illustrated in Figures 6C, D. AMPK can be involved in the metabolic regulation of skeletal muscle via activating its downstream target proteins. It plays an important role in regulating skeletal muscle development through its effects on the cellular anabolism and catabolism processes (Thomson, 2018). FoxO signaling pathway is an important pathway involved in skeletal muscle atrophy (García-Prat et al., 2020). It can be suggested that these DEGs may play a crucial role in the skeletal muscle development of Hu sheep.

### 3.7 Conjoint analysis of differential m6A modification and DEGs

**FIGURE 7:**
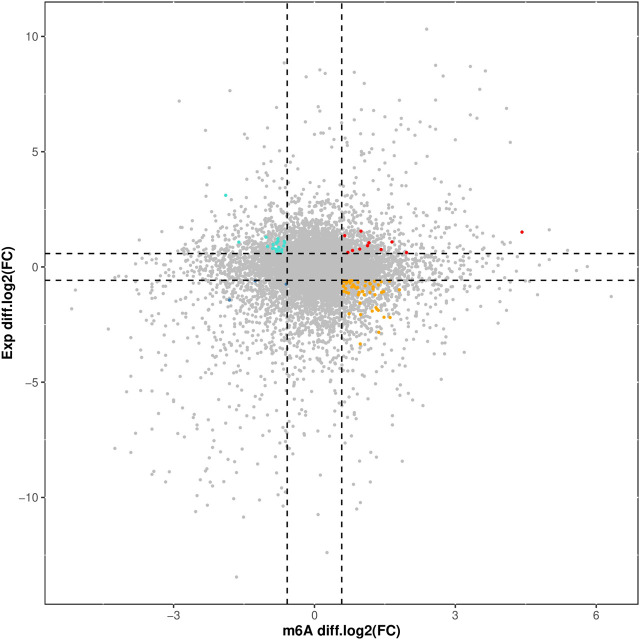
Correlation Analysis of Differential m6A Modification and DEGs.

The overlapping relationship between differential m6A modification-associated genes and DEGs was analyzed based on the two sequencing results (as shown in Figure 7). It was found that there were 283 genes with m6A methylation modification and significant differential expression, among which there were 11 genes with “m6A_up” and “mRNA_up, 20 genes with m6A_down” and “mRNA_up, 3 genes with m6A_down”, and “mRNA_down genes, and 54 genes with m6A_up” and “mRNA_down.”

### 3.8 Validation of DEGs

With the purpose of examining the regulation of skeletal muscle development, three candidate genes, RGMB, MAPK8IP3, and RSPO3, were selected for qRT-PCR validation from relevant genes that changed at both m6A and mRNA levels. These three genes were downregulated in m6A modification but upregulated in mRNA expression (Table 3).

**TABLE 3:** m6A-modified genes related to skeletal muscle development.

Gene name	M6A regulation	Gene regulation	TPM. b_B			TPM. s_B		
			b_B_1	b_B_2	b_B_3	s_B_1	s_B_2	s_B_3
RGMB	Down	up	36.8972	47.6804	41.1691	16.2684	13.3954	9.7801
MAPK8IP3	Down	up	6.7936	6.0416	6.6675	2.9617	2.9609	2.7980
RSPO3	Down	up	13.2870	6.2518	8.8566	3.6945	3.7751	1.5808

**FIGURE 8:**
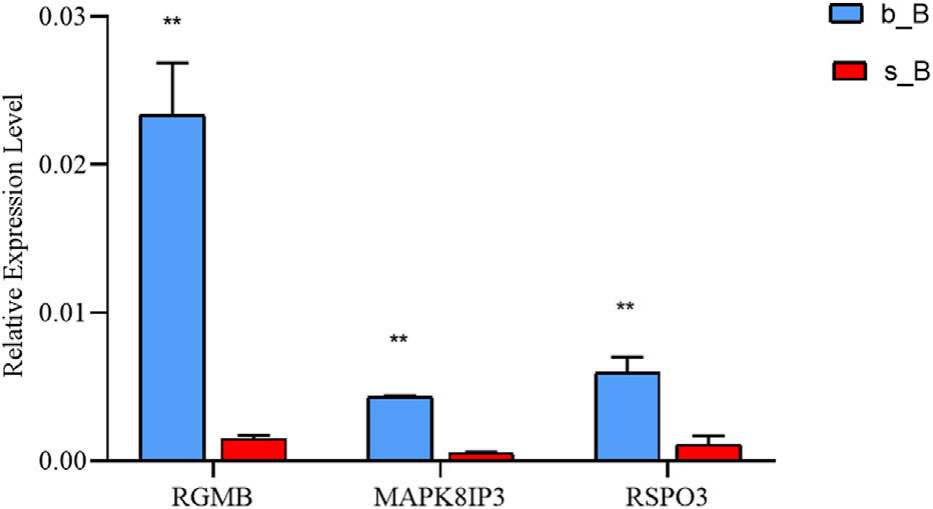
qRT-PCR results of three differential genes in b_B Group and s_B Group.

The qRT-PCR results revealed that the mRNA expression of the three genes in the b_B Group was significantly higher than that of the s_B Group (Figure 8). The changing trends of the three genes were consistent with the RNA-seq results.

## 4 Discussions

In addition to being extensively involved in the regulation of biological processes like disease genesis, embryonic development, and cellular proliferation and differentiation (Zhang et al., 2017; Mendel et al., 2018; Lee et al., 2019; Wang et al., 2020), m6A methylation modifications can also regulate biological processes like RNA shearing, mRNA transporter, mRNA stability, translation, and miRNA processing (Fustin et al., 2013; Geula et al., 2015; Haussmann et al., 2016; Coots et al., 2017). A number of methyltransferases (writers) complexes are primarily responsible for catalyzing the formation of m6A mRNA methylation, which are removed by m6A demethylases (erasers) (Narayan and Rottman, 1988; Bokar et al., 1997), recognized and functional by m6A binding proteins (readers) (Zhao et al., 2021).

Knowledge of skeletal muscle development is essential to unravel the molecular mechanisms of skeletal muscle formation and disease (Buckingham, 2001; Al-Qusairi and Laporte, 2011). In this study, 285 differentially methylated peaks were found in the skeletal muscles of Hu sheep with different months of age, and the conjoint analysis identified 283 genes with m6A methylation modification and significant differential expression. Dou et al. (2023) found 613 differentially methylated peaks between the QA group (3 days of age) and QN (270 days of age) group of Queshan black pigs when investigating their longissimus dorsi muscle. They also found 88 genes with m6A methylation modification and significant differential expression through conjoint analysis. The results show that IGF1R, CCND, MYOD1, FOS, PHKB, BIN1, and FUT2 were involved in the pathways related to muscle development, may play a regulatory role in the process of muscle growth and development. The results of this study can lay a foundation for further determining the potential effect of m6A RNA modification on the regulation of muscle growth of Queshan Black pig. Chen et al. (2022) found 355 differentially methylated peaks in the skeletal muscles of Mountain ducks between the E13 group (Embryo Day 13) and E19 group (Embryo Day 19). They also found 84 genes with m6A methylation modification and significant differential expression through conjoint analysis. They speculate that m6A modification may affect duck muscle development by modulating PDK4, MYLK2 and FBXO40 expression. Xu et al. (2021) found 418 differentially methylated peaks in the skeletal muscles of Dingan goose between the E21 group (Embryo Day 21) and E30 group (Embryo Day 30). Among the m6A-miRNA-genes, they found 10 genes (PDK3, PITPNA, DSTN, BACE, GATM, ITM2A, SOD2, IGFBP4, GAA and TUBB6) are related to breast muscle development which were tightly associated with breast muscle development through affecting the m6A modification levels of their target gene. Wang et al. (2024) found 6,476 differentially methylated peaks in the representatives of leaner Xinghua chicken (XH) and hypertrophic White Recessive Rock chicken (WRR) broilers.They also found 167 genes with m6A methylation modification and significant differential expression through conjoint analysis. As a demethylase of m6A, the highly expression of ALKBH5 in the muscle tissue of poultry and differential expression between XH and WRR chickens suggest that ALKBH5 may play a crucial role in regulating muscle development.

In this study, three candidate genes, RGMB, MAPK8IP3, and RSPO3, were selected for qRT-PCR validation from relevant genes that changed at both m6A and mRNA levels. Results revealed that the mRNA expression of the three genes in the b_B Group was significantly higher than that of the s_B Group, and the changing trends of the three genes were consistent with the RNA-seq results. These results suggested that that RGMB, MAPK8IP3, and RSPO3 play important roles in the skeletal muscle development of Hu sheep. RGMB is the member b of the repulsive guidance molecule (RGM) family (Sartori and Sandri, 2015). Bonemorphogenetic protein (BMP) is a secreted signaling factor belonging to the transforming growth factor β (TGFβ) superfamily, which plays an important role in bone and cartilage formation (Sartori and Sandri, 2015). RGMB regulates a variety of physiological processes mainly through neogenin-Rho and BMP signaling pathways, and it can promote the activation of the signaling pathways (Samad et al., 2005). MAPK8IP3 (mitogen-activated protein kinase 8 interacting protein 3), also known as JIP3 (JNK-interacting protein3), is a JNK (c-Jun N-terminal kinase) scaffold protein (Akechi et al., 2001; Iwasawa et al., 2019). JIP3 generates a complex with JNK and its upstream signaling kinases that promotes the transduction of the JNK signaling pathway (Kelkar et al., 2000; Matsuura et al., 2002; Song and Lee, 2007). Knockdown of JIP3 significantly reduced IR-induced JNK signaling activation and apoptosis (Xu et al., 2010). R-spondin 3 (RSPO3) is a secreted protein belonging to the RSPO family (RSPO1-4) with a common structure of thrombospondin type 1 domain and N-terminal cysteine-rich region. The RSPO family is expressed in normal human placental, lung and muscle tissues (Kim et al., 2006; Kim et al., 2008), which regulates cell proliferation and differentiation by activating the Wnt signaling pathway and is critical for the growth of bone, muscle, blood vessels, and other tissues (Gu et al., 2020). In sheep, there is no report about regulatory role of N6-Methyladenosine in skeletal muscle Development. This study found that m6A plays a certain role in the skeletal muscle development in Hu sheep and the genes RSPO3, RGMB, MAPK8IP33 play important roles in the skeletal muscle development and differentiation. Subsequently, the three candidate genes RSPO3, RGMB, and MAPK8IP33 can be knocked out, knocked down, overexpressed, etc. Finally, Western blot, qPCR, RNA pull down and other detection and verification are carried out to study role and mechanism.

## 5 Conclusion

The findings of this study indicate that the genes RSPO3, RGMB, and MAPK8IP33 may play important roles in the skeletal muscle development and differentiation of Hu sheep. It is inferred that m6A modification has a critical impact on the skeletal muscle development in Hu sheep.

## Data Availability

The datasets presented in this study can be found in online repositories. This data can be found deposited in the “SRA” repository, accession number PRJNA1146914: https://www.ncbi.nlm.nih.gov/bioproject/PRJNA1146914/.

## References

[B1] Akechi M. Ito M. Uemura K. Takamatsu N. Yamashita S. Uchiyama K. (2001). Expression of JNK cascade scaffold protein JSAP1 in the mouse nervous system. Neurosci. Res. 39 (4), 391–400. 10.1016/s0168-0102(01)00194-8 11274738

[B2] Alcaraz-López O. A. Villarreal-Morales Y. Rangel-Escareño C. Gutiérrez-Pabello J. A. (2020). Assessment of candidate biomarkers to detect resistance to Mycobacterium bovis in Holstein-Friesian cattle. Res. Vet. Sci. 132, 416–425. 10.1016/j.rvsc.2020.07.016 32768870

[B3] Al-Qusairi L. Laporte J. (2011). T-tubule biogenesis and triad formation in skeletal muscle and implication in human diseases. Skelet. Muscle 1 (1), 26. 10.1186/2044-5040-1-26 21797990 PMC3156648

[B4] Bokar J. A. Shambaugh M. E. Polayes D. Matera A. G. Rottman F. M. (1997). Purification and cDNA cloning of the AdoMet-binding subunit of the human mRNA (N6-adenosine)-methyltransferase. Rna 3 (11), 1233–1247. 9409616 PMC1369564

[B5] Buckingham M. (2001). Skeletal muscle formation in vertebrates. Curr. Opin. Genet. Dev. 11 (4), 440–448. 10.1016/s0959-437x(00)00215-x 11448631

[B6] Chen B. Liu S. Zhang W. Xiong T. Zhou M. Hu X. (2022). Profiling analysis of N6-methyladenosine mRNA methylation reveals differential m6A patterns during the embryonic skeletal muscle development of ducks. Anim. (Basel) 12 (19), 2593. 10.3390/ani12192593 PMC9559603 36230334

[B7] Coots R. A. Liu X. M. Mao Y. Dong L. Zhou J. Wan J. (2017). m(6)A Facilitates eIF4F-Independent mRNA Translation. Mol. Cell 68 (3), 504–514. 10.1016/j.molcel.2017.10.002 29107534 PMC5913006

[B8] Deng K. Fan Y. Liang Y. Cai Y. Zhang G. Deng M. (2021). FTO-mediated demethylation of GADD45B promotes myogenesis through the activation of p38 MAPK pathway. Mol. Ther. Nucleic Acids 26, 34–48. 10.1016/j.omtn.2021.06.013 34513292 PMC8408560

[B9] Dou Y. Wei Y. Zhang Z. Li C. Song C. Liu Y. (2023). Transcriptome-wide analysis of RNA m(6)A methylation regulation of muscle development in Queshan Black pigs. BMC Genomics 24 (1), 239. 10.1186/s12864-023-09346-w 37142996 PMC10161540

[B10] Fustin J. M. Doi M. Yamaguchi Y. Hida H. Nishimura S. Yoshida M. (2013). RNA-methylation-dependent RNA processing controls the speed of the circadian clock. Cell 155 (4), 793–806. 10.1016/j.cell.2013.10.026 24209618

[B11] García-Prat L. Perdiguero E. Alonso-Martín S. Dell'Orso S. Ravichandran S. Brooks S. R. (2020). FoxO maintains a genuine muscle stem-cell quiescent state until geriatric age. Nat. Cell Biol. 22 (11), 1307–1318. 10.1038/s41556-020-00593-7 33106654

[B12] Geula S. Moshitch-Moshkovitz S. Dominissini D. Mansour A. A. Kol N. Salmon-Divon M. (2015). Stem cells. m6A mRNA methylation facilitates resolution of naïve pluripotency toward differentiation. Science 347 (6225), 1002–1006. 10.1126/science.1261417 25569111

[B13] Gu H. Tu H. Liu L. Liu T. Liu Z. Zhang W. (2020). RSPO3 is a marker candidate for predicting tumor aggressiveness in ovarian cancer. Ann. Transl. Med. 8 (21), 1351. 10.21037/atm-20-3731 33313096 PMC7723610

[B14] Hansen T. B. Venø M. T. Damgaard C. K. Kjems J. (2016). Comparison of circular RNA prediction tools. Nucleic Acids Res. 44 (6), e58. 10.1093/nar/gkv1458 26657634 PMC4824091

[B15] Haussmann I. U. Bodi Z. Sanchez-Moran E. Mongan N. P. Archer N. Fray R. G. (2016). m(6)A potentiates Sxl alternative pre-mRNA splicing for robust Drosophila sex determination. Nature 540 (7632), 301–304. 10.1038/nature20577 27919081

[B16] Heinz S. Benner C. Spann N. Bertolino E. Lin Y. C. Laslo P. (2010). Simple combinations of lineage-determining transcription factors prime cis-regulatory elements required for macrophage and B cell identities. Mol. Cell 38 (4), 576–589. 10.1016/j.molcel.2010.05.004 20513432 PMC2898526

[B17] Huang H. Weng H. Chen J. (2020). m(6)A modification in coding and non-coding RNAs: roles and therapeutic implications in cancer. Cancer Cell 37 (3), 270–288. 10.1016/j.ccell.2020.02.004 32183948 PMC7141420

[B18] Iwasawa S. Yanagi K. Kikuchi A. Kobayashi Y. Haginoya K. Matsumoto H. (2019). Recurrent *de novo* MAPK8IP3 variants cause neurological phenotypes. Ann. Neurol. 85 (6), 927–933. 10.1002/ana.25481 30945334

[B19] Kelkar N. Gupta S. Dickens M. Davis R. J. (2000). Interaction of a mitogen-activated protein kinase signaling module with the neuronal protein JIP3. Mol. Cell Biol. 20 (3), 1030–1043. 10.1128/mcb.20.3.1030-1043.2000 10629060 PMC85220

[B20] Kim D. Langmead B. Salzberg S. L. (2015). HISAT: a fast spliced aligner with low memory requirements. Nat. Methods 12 (4), 357–360. 10.1038/nmeth.3317 25751142 PMC4655817

[B21] Kim K. A. Wagle M. Tran K. Zhan X. Dixon M. A. Liu S. (2008). R-Spondin family members regulate the Wnt pathway by a common mechanism. Mol. Biol. Cell 19 (6), 2588–2596. 10.1091/mbc.e08-02-0187 18400942 PMC2397303

[B22] Kim K. A. Zhao J. Andarmani S. Kakitani M. Oshima T. Binnerts M. E. (2006). R-Spondin proteins: a novel link to beta-catenin activation. Cell Cycle 5 (1), 23–26. 10.4161/cc.5.1.2305 16357527

[B23] Lee H. Bao S. Qian Y. Geula S. Leslie J. Zhang C. (2019). Stage-specific requirement for Mettl3-dependent m(6)A mRNA methylation during haematopoietic stem cell differentiation. Nat. Cell Biol. 21 (6), 700–709. 10.1038/s41556-019-0318-1 31061465 PMC6556891

[B24] Liu J. Yue Y. Han D. Wang X. Fu Y. Zhang L. (2014). A METTL3-METTL14 complex mediates mammalian nuclear RNA N6-adenosine methylation. Nat. Chem. Biol. 10 (2), 93–95. 10.1038/nchembio.1432 24316715 PMC3911877

[B25] Ma S. Chen C. Ji X. Liu J. Zhou Q. Wang G. (2019). The interplay between m6A RNA methylation and noncoding RNA in cancer. J. Hematol. Oncol. 12 (1), 121. 10.1186/s13045-019-0805-7 31757221 PMC6874823

[B26] Ma X. La Y. Bao P. Chu M. Guo X. Wu X. (2022). Regulatory role of N6-methyladenosine in longissimus dorsi development in yak. Front. Vet. Sci. 9, 757115. 10.3389/fvets.2022.757115 35498742 PMC9043854

[B27] Matsuura H. Nishitoh H. Takeda K. Matsuzawa A. Amagasa T. Ito M. (2002). Phosphorylation-dependent scaffolding role of JSAP1/JIP3 in the ASK1-JNK signaling pathway. A new mode of regulation of the MAP kinase cascade. J. Biol. Chem. 277 (43), 40703–40709. 10.1074/jbc.M202004200 12189133

[B28] Mendel M. Chen K. M. Homolka D. Gos P. Pandey R. R. McCarthy A. A. (2018). Methylation of structured RNA by the m(6)A writer METTL16 is essential for mouse embryonic development. Mol. Cell 71 (6), 986–1000. 10.1016/j.molcel.2018.08.004 30197299 PMC6162343

[B29] Meng J. Cui X. Rao M. K. Chen Y. Huang Y. (2013). Exome-based analysis for RNA epigenome sequencing data. Bioinformatics 29 (12), 1565–1567. 10.1093/bioinformatics/btt171 23589649 PMC3673212

[B30] Meng J. Lu Z. Liu H. Zhang L. Zhang S. Chen Y. (2014). A protocol for RNA methylation differential analysis with MeRIP-Seq data and exomePeak R/Bioconductor package. Methods 69 (3), 274–281. 10.1016/j.ymeth.2014.06.008 24979058 PMC4194139

[B31] Meyer K. D. Saletore Y. Zumbo P. Elemento O. Mason C. E. Jaffrey S. R. (2012). Comprehensive analysis of mRNA methylation reveals enrichment in 3' UTRs and near stop codons. Cell 149 (7), 1635–1646. 10.1016/j.cell.2012.05.003 22608085 PMC3383396

[B32] Narayan P. Rottman F. M. (1988). An *in vitro* system for accurate methylation of internal adenosine residues in messenger RNA. Science 242 (4882), 1159–1162. 10.1126/science.3187541 3187541

[B33] Pertea M. Kim D. Pertea G. M. Leek J. T. Salzberg S. L. (2016). Transcript-level expression analysis of RNA-seq experiments with HISAT, StringTie and Ballgown. Nat. Protoc. 11 (9), 1650–1667. 10.1038/nprot.2016.095 27560171 PMC5032908

[B34] Samad T. A. Rebbapragada A. Bell E. Zhang Y. Sidis Y. Jeong S. J. (2005). DRAGON, a bone morphogenetic protein co-receptor. J. Biol. Chem. 280 (14), 14122–14129. 10.1074/jbc.M410034200 15671031

[B35] Sartori R. Sandri M. (2015). BMPs and the muscle-bone connection. Bone 80, 37–42. 10.1016/j.bone.2015.05.023 26036170

[B36] Song J. J. Lee Y. J. (2007). Differential activation of the JNK signal pathway by UV irradiation and glucose deprivation. Cell Signal 19 (3), 563–572. 10.1016/j.cellsig.2006.08.016 17029735

[B37] Sorci M. Ianniello Z. Cruciani S. Larivera S. Ginistrelli L. C. Capuano E. (2018). METTL3 regulates WTAP protein homeostasis. Cell Death Dis. 9 (8), 796. 10.1038/s41419-018-0843-z 30038300 PMC6056540

[B38] Thomson D. M. (2018). The role of AMPK in the regulation of skeletal muscle size, hypertrophy, and regeneration. Int. J. Mol. Sci. 19 (10), 3125. 10.3390/ijms19103125 30314396 PMC6212977

[B39] Vorachek W. R. Hugejiletu B. G. Hall J. A. (2013). Reference gene selection for quantitative PCR studies in sheep neutrophils. Int. J. Mol. Sci. 14 (6), 11484–11495. 10.3390/ijms140611484 23722658 PMC3709743

[B40] Wang Q. Chen C. Ding Q. Zhao Y. Wang Z. Chen J. (2020). METTL3-mediated m(6)A modification of HDGF mRNA promotes gastric cancer progression and has prognostic significance. Gut 69 (7), 1193–1205. 10.1136/gutjnl-2019-319639 31582403

[B41] Wang S. Lv W. Li T. Zhang S. Wang H. Li X. (2022). Dynamic regulation and functions of mRNA m6A modification. Cancer Cell Int. 22 (1), 48. 10.1186/s12935-022-02452-x 35093087 PMC8800407

[B42] Wang Z. Ju X. Li K. Cai D. Zhou Z. Nie Q. (2024). MeRIP sequencing reveals the regulation of N6-methyladenosine in muscle development between hypertrophic and leaner broilers. Poult. Sci. 103 (6), 103708. 10.1016/j.psj.2024.103708 38631230 PMC11040168

[B43] Xu B. Zhou Y. O K. Choy P. C. Pierce G. N. Siow Y. L. (2010). Regulation of stress-associated scaffold proteins JIP1 and JIP3 on the c-Jun NH2-terminal kinase in ischemia-reperfusion. Can. J. Physiol. Pharmacol. 88 (11), 1084–1092. 10.1139/y10-088 21076496

[B44] Xu T. Xu Z. Lu L. Zeng T. Gu L. Huang Y. (2021). Transcriptome-wide study revealed m6A regulation of embryonic muscle development in Dingan goose (*Anser cygnoides* orientalis). BMC Genomics 22 (1), 270. 10.1186/s12864-021-07556-8 33853538 PMC8048326

[B45] Yu G. Wang L. G. He Q. Y. (2015). ChIPseeker: an R/Bioconductor package for ChIP peak annotation, comparison and visualization. Bioinformatics 31 (14), 2382–2383. 10.1093/bioinformatics/btv145 25765347

[B46] Zhang S. Zhao B. S. Zhou A. Lin K. Zheng S. Lu Z. (2017). m(6)A demethylase ALKBH5 maintains tumorigenicity of glioblastoma stem-like cells by sustaining FOXM1 expression and cell proliferation program. Cancer Cell 31 (4), 591–606. 10.1016/j.ccell.2017.02.013 28344040 PMC5427719

[B47] Zhang X. Yao Y. Han J. Yang Y. Chen Y. Tang Z. (2020). Longitudinal epitranscriptome profiling reveals the crucial role of N(6)-methyladenosine methylation in porcine prenatal skeletal muscle development. J. Genet. Genomics 47 (8), 466–476. 10.1016/j.jgg.2020.07.003 33268291

[B48] Zhao L. Yuan L. Li F. Zhang X. Tian H. Ma Z. (2024). Whole-genome resequencing of Hu sheep identifies candidate genes associated with agronomic traits. J. Genet. Genomics 51, 866–876. 10.1016/j.jgg.2024.03.015 38582298

[B49] Zhao Y. Chen Y. Jin M. Wang J. (2021). The crosstalk between m(6)A RNA methylation and other epigenetic regulators: a novel perspective in epigenetic remodeling. Theranostics 11 (9), 4549–4566. 10.7150/thno.54967 33754077 PMC7977459

