# Supplementary material for: Regulatory role of N6-Methyladenosine on skeletal muscle development in Hu sheep
Source: Front Genet. 2024 Aug 21;15:1449144. doi: 10.3389/fgene.2024.1449144 (PMC11371687; doi:10.3389/fgene.2024.1449144)
Supplement: Supplementary file 1 [file Table3.DOC]

Table S3 Primer List

| Primer name | Sequence(5'-3') | Product Size/bp |
| --- | --- | --- |
| RGMB-F | GATGGCAATGGGTAAGGAG | 177 |
| RGMB-R | CAAATCACAGAGGGACGAG |  |
| MAPK8IP3-F | TAGGGGAGTTCTCAGTGCGT | 104 |
| MAPK8IP3-R | GTTCAGAGCGTTTTTGGTTT |  |
| RSPO3-F | GGAAGCCAATAACCACACC | 164 |
| RSPO3-R | TACCCTTTGCTGACGGATG |  |
| GAPDH-F | CATGTTTGTGATGGGCGTGAAC | 303 |
| GAPDH-R | CCAGTGAGCTTCCCGTTGA |  |
