# Supplementary material for: Regulatory role of N6-Methyladenosine on skeletal muscle development in Hu sheep
Source: Front Genet. 2024 Aug 21;15:1449144. doi: 10.3389/fgene.2024.1449144 (PMC11371687; doi:10.3389/fgene.2024.1449144)
Supplement: Supplementary file 2 [file Table1.DOC]

TableS1 Summary of reads quality control

| SampleID | RawReadNum | RawBaseNum | RawQ20 | RawQ30 | CleanReadNum | CleanBaseNum | CleanQ20 | CleanQ30 | CleanRate | CleanData(GB) |
| --- | --- | --- | --- | --- | --- | --- | --- | --- | --- | --- |
| b_B_1_IP | 109734572 | 16460185800 | 97.19% | 93.34% | 109604864 | 15008489987 | 97.60% | 93.77% | 91.18% | 15.01 |
| b_B_1_Input | 150078922 | 22511838300 | 97.06% | 92.91% | 149706464 | 19857662600 | 97.44% | 93.37% | 88.21% | 19.86 |
| b_B_2_IP | 145330396 | 21799559400 | 96.54% | 92.45% | 145130626 | 19452218363 | 97.27% | 93.23% | 89.23% | 19.45 |
| b_B_2_Input | 136065048 | 20409757200 | 96.82% | 92.53% | 135789832 | 18288013295 | 97.19% | 92.97% | 89.60% | 18.29 |
| b_B_3_IP | 122748308 | 18412246200 | 97.19% | 93.30% | 122595020 | 16486264739 | 97.69% | 93.84% | 89.54% | 16.49 |
| b_B_3_Input | 146529998 | 21979499700 | 96.94% | 92.73% | 146178602 | 19633937922 | 97.34% | 93.22% | 89.33% | 19.63 |
| s_B_1_IP | 102211158 | 15331673700 | 97.40% | 93.69% | 102111742 | 13901430333 | 97.84% | 94.15% | 90.67% | 13.9 |
| s_B_1_Input | 142069992 | 21310498800 | 97.22% | 93.04% | 141707514 | 19271474679 | 97.53% | 93.38% | 90.43% | 19.27 |
| s_B_2_IP | 108422506 | 16263375900 | 97.06% | 93.05% | 108320674 | 14786812817 | 97.51% | 93.52% | 90.92% | 14.79 |
| s_B_2_Input | 128223112 | 19233466800 | 97.34% | 93.36% | 127861086 | 17068902275 | 97.68% | 93.77% | 88.75% | 17.07 |
| s_B_3_IP | 126578068 | 18986710200 | 97.00% | 93.22% | 126446894 | 17139390618 | 97.55% | 93.81% | 90.27% | 17.14 |
| s_B_3_Input | 118312186 | 17746827900 | 97.50% | 93.71% | 117937062 | 15461457346 | 97.86% | 94.18% | 87.12% | 15.46 |
