# Supplementary material for: Regulatory role of N6-Methyladenosine on skeletal muscle development in Hu sheep
Source: Front Genet. 2024 Aug 21;15:1449144. doi: 10.3389/fgene.2024.1449144 (PMC11371687; doi:10.3389/fgene.2024.1449144)
Supplement: Supplementary file 3 [file Table2.DOC]

TableS2 Summary of reads mapped to the Sheep

| Sample | Mapped_Reads | Map_Rate | Uniq_Reads | Uniq_Rate |
| --- | --- | --- | --- | --- |
| b_B_1_Input | 143031655 | 95.54% | 108485449 | 72.47% |
| b_B_1_IP | 104364230 | 95.22% | 80497715 | 73.44% |
| b_B_2_Input | 128119988 | 94.35% | 96063886 | 70.74% |
| b_B_2_IP | 137137051 | 94.49% | 103404201 | 71.25% |
| b_B_3_Input | 138694095 | 94.88% | 106330201 | 72.74% |
| b_B_3_IP | 117427335 | 95.78% | 91858890 | 74.93% |
| s_B_1_Input | 122358012 | 86.35% | 93820019 | 66.21% |
| s_B_1_IP | 94448010 | 92.49% | 71539346 | 70.06% |
| s_B_2_Input | 96257977 | 75.28% | 71570706 | 55.98% |
| s_B_2_IP | 93886477 | 86.67% | 70833446 | 65.39% |
| s_B_3_Input | 87789672 | 74.44% | 64158548 | 54.40% |
| s_B_3_IP | 108514891 | 85.82% | 80465766 | 63.64% |
